# Supplementary material for: Foxp1 controls brown/beige adipocyte differentiation and thermogenesis through regulating β3-AR desensitization
Source: Nat Commun. 2019 Nov 7;10:5070. doi: 10.1038/s41467-019-12988-8 (PMC6838312; doi:10.1038/s41467-019-12988-8)
Supplement: Supplementary file 1 — Supplementary Information [file 41467_2019_12988_MOESM1_ESM.pdf]

Supplementary Information

Foxp1 controls brown/beige adipocyte differentiation and thermogenesis through regulating β3-AR desensitization

Liu., et al.,

Supplementary Figures

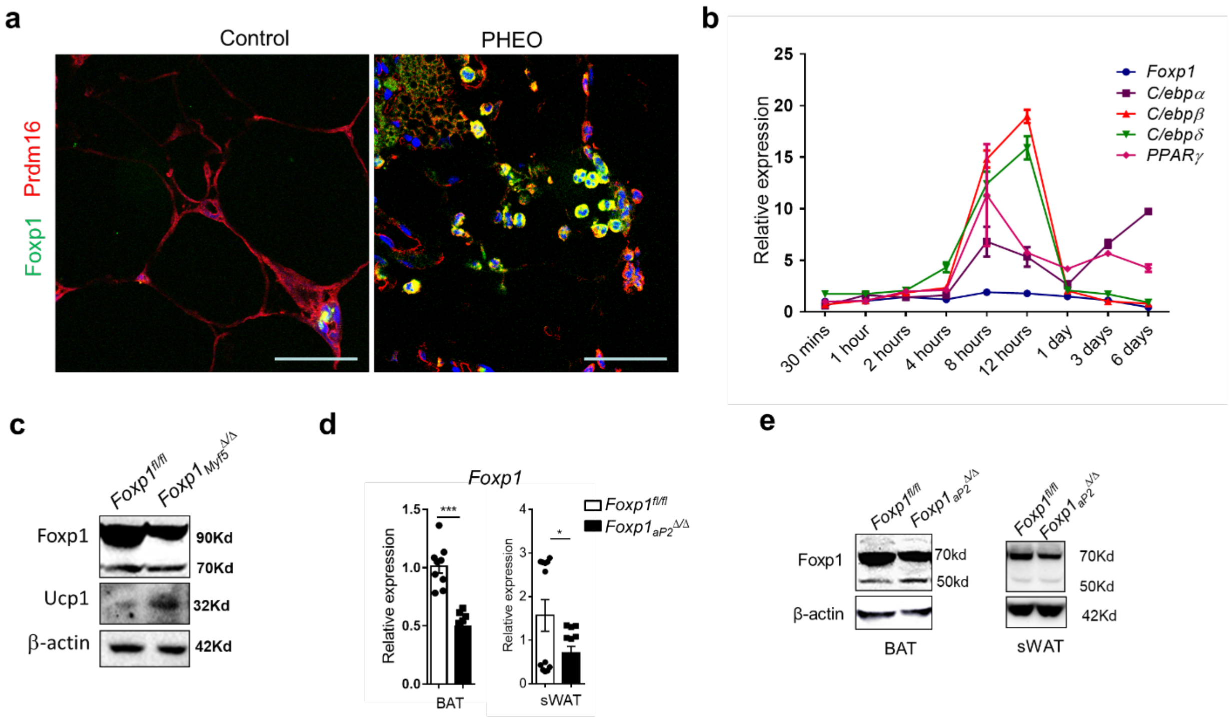

Supplementary Figure 1 Expression of *Foxp1* in adipose tissues from knockout mice. **a** Immunofluorescence staining for sections from PHEO biopsies with Foxp1 and Prdm16 antibodies. Bar, 50μm. **b** qPCR showed the expression of *Foxp1* in the time course of adipogenesis in 3T3-L1 cell line. n=3 biologically independent samples. **c** Western blotting for the Foxp1 and Ucp1 expression in BAT from $Foxp1_{Myf5}^{\Delta/\Delta}$ at P7. **d** qPCR for the *Foxp1* expression in BAT and sWAT from $Foxp1_{aP2}^{\Delta/\Delta}$ mice. n=3 biologically independent samples. **e** Western blotting for the Foxp1 expression in BAT and sWAT from $Foxp1_{aP2}^{\Delta/\Delta}$ mice at age of 2 months. *, P<0.05; ***, P<0.001; error bar, mean±SEM.

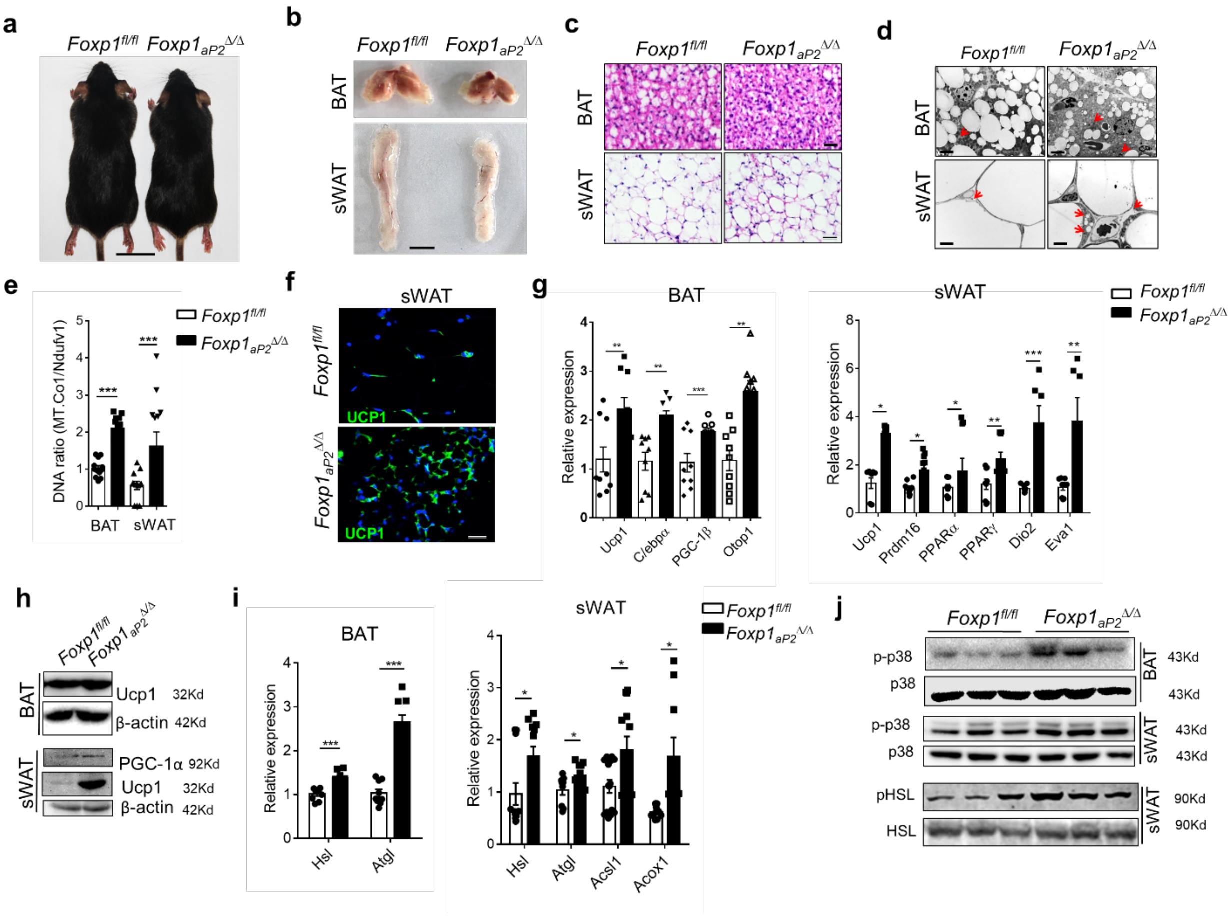

Supplementary Figure 2 Adipose Tissue Browning in *Foxp1*$_{aP2}^{\Delta/\Delta}$ mice.

a Representative dorsal view of *Foxp1*$^{fl/fl}$ and *Foxp1*$_{aP2}^{\Delta/\Delta}$ mice at age of 8 weeks. Bar, 2cm. **b** Overview of BAT and sWAT depot in mice from (**a**). Bar, 1cm. **c** Representative pictures of HE staining for BAT and sWAT. Bar, 10μm. **d** Intracellular structure of adipocytes in BAT and sWAT from mice, as showed by TEM. Bar, 2μm. Red arrows, mitochondria. **e** Abundance of mitochondria DNA was evaluated by relative expression of mitochondrial gene *Co1* in BAT and sWAT. n=6 biologically independent samples. **f** Representative immunofluorescence staining for sWAT with anti-UCP1. Bar, 10μm. **g** qPCR of BAT-selective markers in BAT and sWAT. n=3 biologically independent samples. **h** Western blotting with UCP1 and PGC1α antibody in BAT or sWAT. **i** qPCR for lipolytic genes in BAT and sWAT. n=3 biologically independent samples. **j** Western blotting for p38, phosphorylated p38, HSL and phosphorylated HSL in BAT and sWAT. *, $P<0.05$, **, $P<0.01$, ***, $P<0.001$; error bar, mean±SEM.

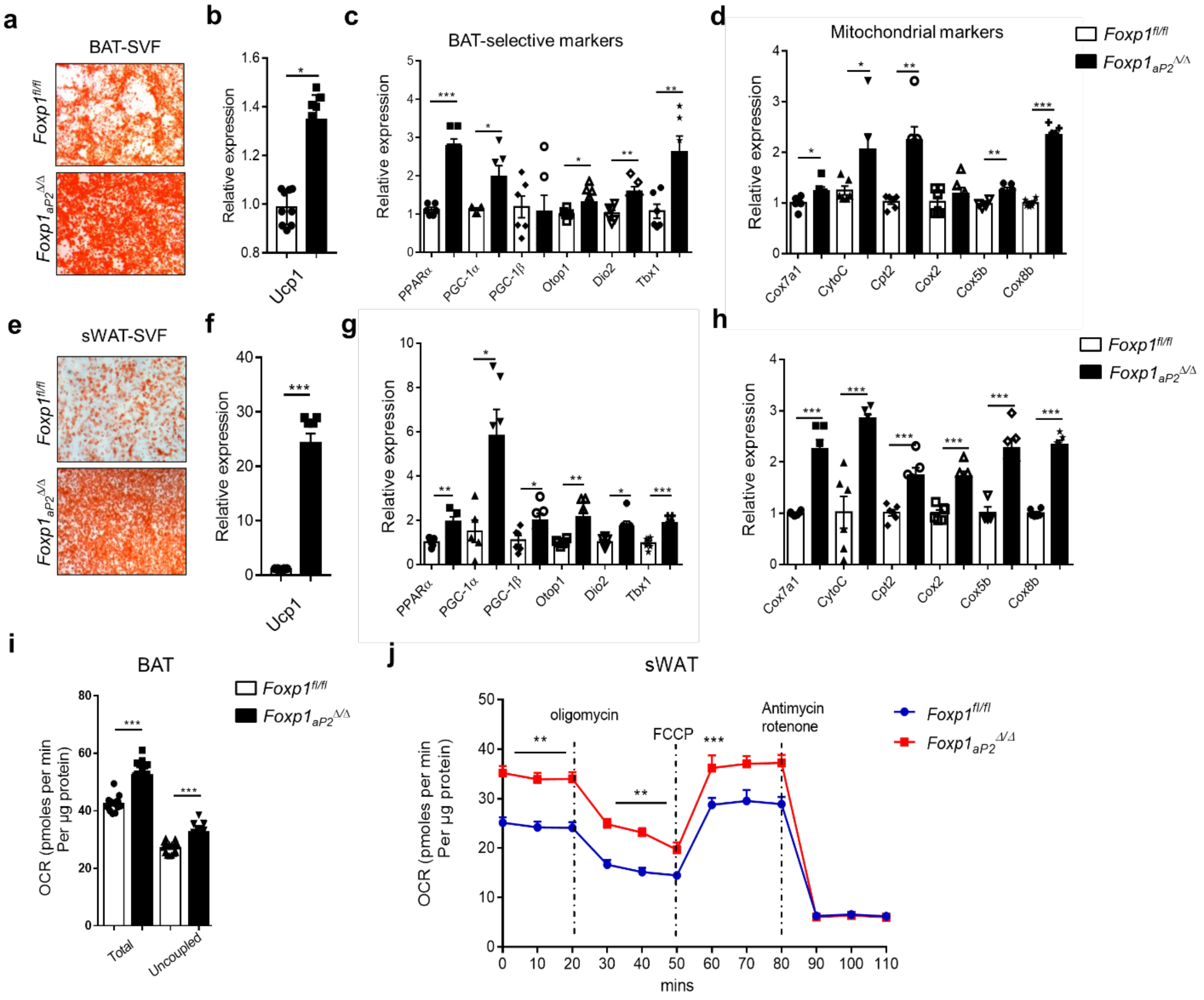

Supplementary Figure 3 *Foxp1* deletion potentiates brown adipocyte differentiation *in vitro*.

a Oil Red O staining for 8-day brown adipocyte differentiation of SVF isolated from BAT of *Foxp1*$^{fl/fl}$ and *Foxp1*$_{aP2}^{\Delta/\Delta}$ mice at age of 8 weeks. **b-d** qPCR of BAT-selective and mitochondrial markers in brown adipocytes in (**a**). **e** Oil Red O staining for beige adipocytes stemmed from SVF in sWAT. **f-h** qPCR of BAT-selective and mitochondrial markers in brown adipocytes in (**e**). **i, j** Oxygen consumption rate was measured for adipocytes from BAT (**i**) and sWAT (**j**). Uncoupled respiration was recorded after oligomycin inhibition of ATP synthesis, and maximal respiration following stimulation with FCCP. n=6 biologically independent samples; **, P<0.01; ***, P<0.001; error bar, mean±SEM.

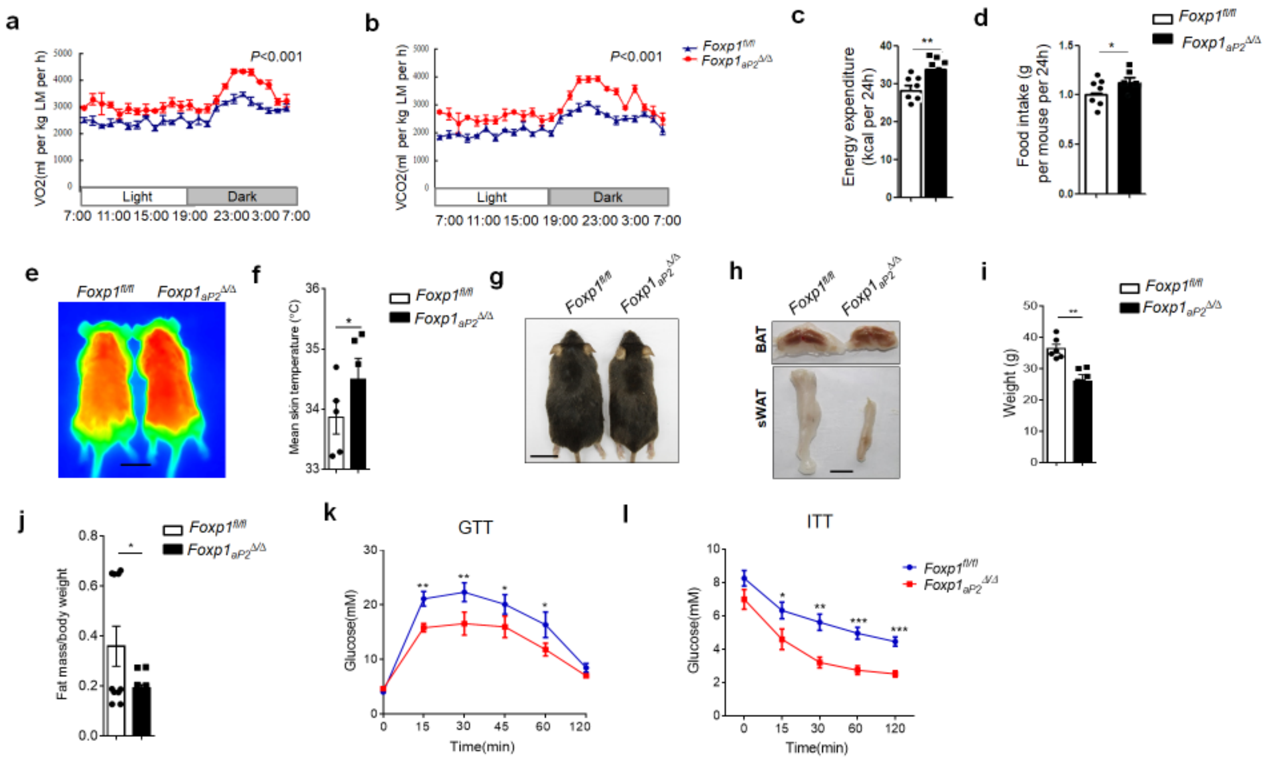

Supplementary Figure 4 Energy metabolism in *Foxp1*$_{aP2}^{\Delta/\Delta}$ mice.

a, b Consumption of O_2 (**a**) and CO_2 (**b**) of *Foxp1*$^{fl/fl}$ and *Foxp1*$_{aP2}^{\Delta/\Delta}$mice at age of 8 weeks old. **c, d** Energy expenditure (**c**) and food intake (**d**) in mice. n=8 biologically independent mice/each group. **e** Representative infrared imaging of mice after one-hour 4℃ exposure. Bar, 2cm. **f** Mean dorsal skin temperature in (**e**). n=5 biologically independent mice/each group. **g** Dorsal view of mice fed with HFD for 16 weeks starting at age of 10 weeks old. Bar, 2cm. **h** Comparative view of adipose depots of BAT and sWAT in mice in (**g**). Bar, 1cm. **i, j** Body weight and adiposity were measured for HFD-fed mice. **k, l** GTT and ITT tests for HFD-fed mice. n=8 biologically independent mice/each group. *, P<0.05; **, P<0.01; ***, P<0.001; error bar, mean±SEM.

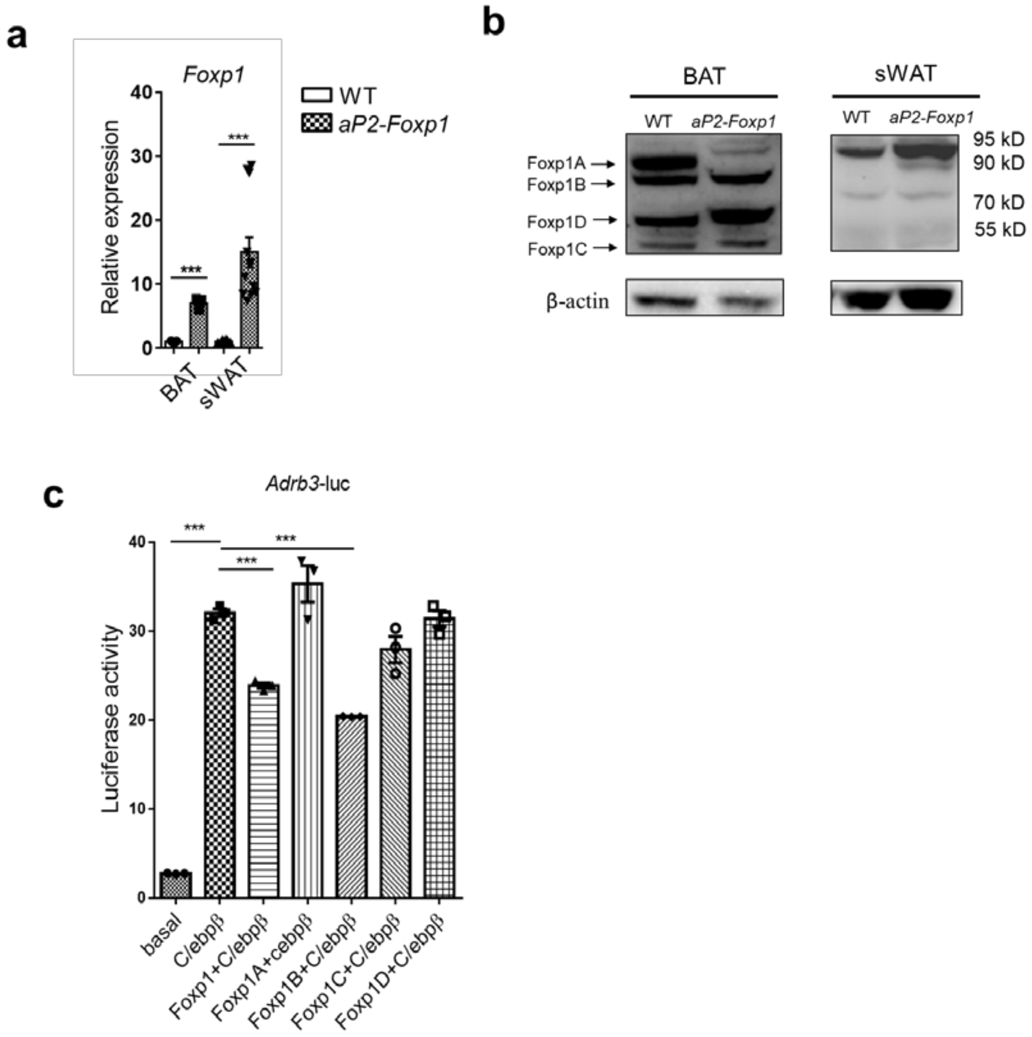

Supplementary Figure 5 Overexpression of *Foxp1* in transgenic mice.

a qPCR analysis showed the expression of *Foxp1* in BAT and sWAT in *aP2-Foxp1* transgenic mice at age of 2 months. **b** Western blotting for the Foxp1 expression in transgenic mice (**a**). **c** Luciferase reporter assay in 293T cells, which were transfected with *Adrb3*-Luc, C/ebpβ and Foxp1, Foxp1A-D expression vectors. ***, *P*<0.001; n=3 biologically independent experiments; error bar, mean±SEM.

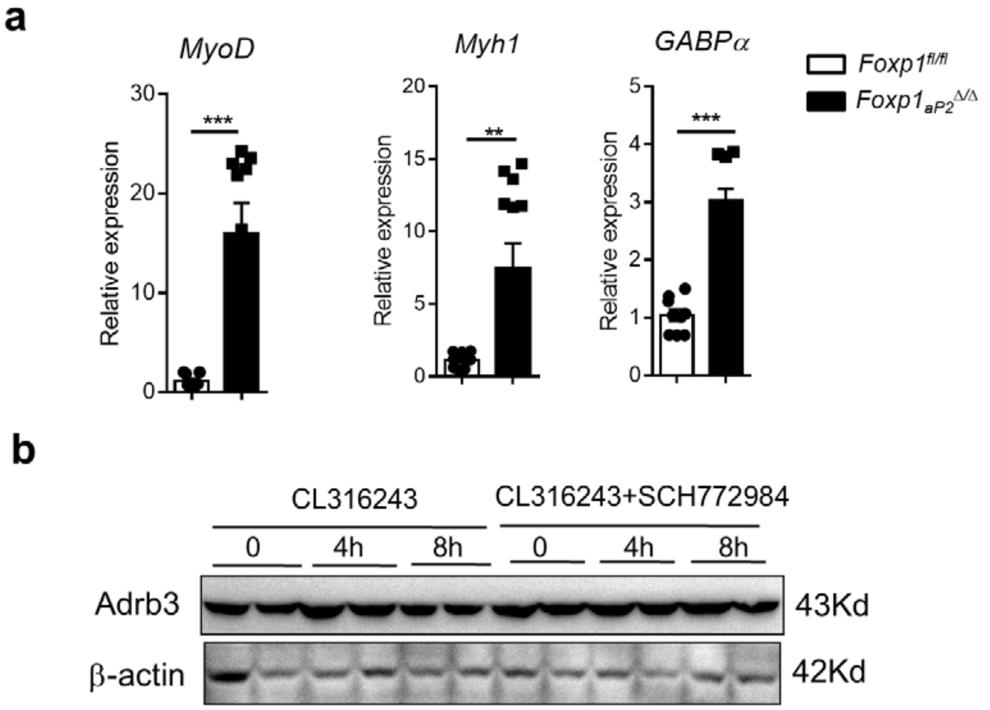

Supplementary Figure 6 The impact of *Foxp1* deletion on β-AR-independent beige induction.

a qPCR for glycolytic beige related marker genes in WAT from *Foxp1*$_{aP2}^{\Delta/\Delta}$mice at age of 8 weeks old. **, *P*<0.01; ***, *P*<0.001; n=3 biologically independent mice/each group; error bar, mean±s.e.m. **b** Western blotting for Adrb3 (β3-AR) in brown adipocytes derived from SVF, which were stimulated by CL-316,243 (0.1 μM) with or without SCH772984 (10 μM) for 8 hours.

Supplementary Table 1

Foxp1 ChIP-seq BAT selective targets

Coordinate	Chr	Start	End	Strand	Gene Name	Gene Alias	Detailed Annotation	Distance to TSS
chr5:51786580-51786745	chr5	51786580	51786745	+	Ppargc1a	A830037N07Rik|Gm11133|PGC-1|PPARGC-1-alpha|Pgc-1alpha|Pgc1|Pgco1|Ppargc1	Intergenic	-218937
chr5:51778192-51778424	chr5	51778192	51778424	+	Ppargc1a	A830037N07Rik|Gm11133|PGC-1|PPARGC-1-alpha|Pgc-1alpha|Pgc1|Pgco1|Ppargc1	Intergenic	-210583
chr5:51032977-51033142	chr5	51032977	51033142	+	Ppargc1a	A830037N07Rik|Gm11133|PGC-1|PPARGC-1-alpha|Pgc-1alpha|Pgc1|Pgco1|Ppargc1	Intergenic	520862
chr5:50923530-50923695	chr5	50923530	50923695	+	Ppargc1a	A830037N07Rik|Gm11133|PGC-1|PPARGC-1-alpha|Pgc-1alpha|Pgc1|Pgco1|Ppargc1	Intergenic	630309
chr5:50976524-50976689	chr5	50976524	50976689	+	Ppargc1a	A830037N07Rik|Gm11133|PGC-1|PPARGC-1-alpha|Pgc-1alpha|Pgc1|Pgco1|Ppargc1	CT-rich|Low_complexity|Low_complexity	577315
chr5:50939045-50939210	chr5	50939045	50939210	+	Ppargc1a	A830037N07Rik|Gm11133|PGC-1|PPARGC-1-alpha|Pgc-1alpha|Pgc1|Pgco1|Ppargc1	(CAAAAA)n|Simple_repeat|Simple_repeat	614794
chr6:115425204-115425418	chr6	115425204	115425418	+	Pparg	Nr1c3|PPAR-gamma|PPAR-gamma2|PPARgamma|PPARgamma2	intron (NM_011146, intron 1 of 6)	3245
chr12:34003367-34003532	chr12	34003367	34003532	+	Twist1	M-Twist|Pde|Ska10|Ska<m10Jus>|Twist|bHLHa38|pdt	A-rich|Low_complexity|Low_complexity	45778
chr12:34197983-34198148	chr12	34197983	34198148	+	Twist1	M-Twist|Pde|Ska10|Ska<m10Jus>|Twist|bHLHa38|pdt	B1_Mus2|SINE|Alu	240394
chr12:34110267-34110513	chr12	34110267	34110513	+	Twist1	M-Twist|Pde|Ska10|Ska<m10Jus>|Twist|bHLHa38|pdt	intron (NM_001271386, intron 24 of 27)	152719
chr12:90384896-90385061	chr12	90384896	90385061	+	Dio2	5DII|AI324267|DIOII	L1_Mus1|LINE|L1	354059
chr12:90460056-90460221	chr12	90460056	90460221	+	Dio2	5DII|AI324267|DIOII	Intergenic	278899

chr12:90803437-90803602	chr12	90803437	90803602	+	Dio2	5DII|AI324267|DIOII	Intergenic	-64482
chr12:90400496-90400661	chr12	90400496	90400661	+	Dio2	5DII|AI324267|DIOII	MTE2b|LTR|ERVL-MaLR	338459
chr12:90384896-90385061	chr12	90384896	90385061	+	Dio2	5DII|AI324267|DIOII	L1_Mus1|LINE|L1	354059
chr12:90460056-90460221	chr12	90460056	90460221	+	Dio2	5DII|AI324267|DIOII	Intergenic	278899
chr12:90803437-90803602	chr12	90803437	90803602	+	Dio2	5DII|AI324267|DIOII	Intergenic	-64482
chr8:91714875-91715183	chr8	91714875	91715183	+	Irx3	AI894186	Intergenic	86625
chr4:107936778-107936973	chr4	107936778	107936973	+	Cpt2	AI323697|CPTII	Intergenic	-13286
chr4:107937564-107937729	chr4	107937564	107937729	+	Cpt2	AI323697|CPTII	Intergenic	-14057

Supplementary Table 2

Sequence of oligos for genetyping, qPCR and shRNA construct

genes	qPCR	sequence
Foxp1	F	TCTCGTCCTCGGCACCTT
	R	GTCACAAACCGCCTCACA
Adrb3	F	GGCCCTCTCTAGTTCCCAG
	R	TAGCCATCAAACCTGTTGAGC
Ucp1	F	ACTGCCACACCTCCAGTCATT
	R	CTTTGCCTCACTCAGGATTGG
PGC-1α	F	AGCCGTGACCACTGACAACGAG
	R	GCTGCATGGTTCTGAGTGCTAAG
PGC-1β	F	CTCCAGGCAGGTTCAACCC
	R	GGGCCAGAAGTTCCCTTAGG
Fabp4	F	GATGAAATCACCGCAGACGACA
	R	ATTGTGGTCGACTTTCCATCCC
C/ebpα	F	TGGACAAGAACAGCAACGAG
	R	TCACTGGTCAACTCCAGCAC
C/ebpβ	F	ACGACTTCCTCTCCGACCTCT
	R	CGAGGCTCACGTAACCGTAGT
Dio2	F	CAGTGTGGTGCACGTCTCCAATC
	R	TGAACCAAAGTTGACCACCAG
Prdm16	F	CCACCAGCGAGGACTTCAC
	R	GGAGGACTCTCGTAGCTCGAA
Cox2	F	GCAAGCATAAGACTGGACCAAA
	R	TTGTTGGCATCTGTGTAAGAGAATC
Cox4il	F	ACCAAGCGAATGCTGGACAT
	R	GGCGGAGAAGCCCTGAA
β-actin	F	AGAGGGAAATCGTGCGTGACA
	R	CACTGTGTTGGCATAGAGGTC
Tbx1	F	GGCAGGCAGACGAATGTTC
	R	TTGTCATCTACGGGCACAAAG
Elovl3	F	TCCGCGTTCTCATGTAGGTCT
	R	GGACCTGATGCAACCCTATGA
Cyt C	F	TCCATCAGGGTATCCTCTCC
	R	GGAGGCAAGCAGAAGACTGG
Cox5b	F	GCTGCATCTGTGAAGAGGACAAC
	R	CAGCTTGTAATGGGTTCCACAGT
Cox8b	F	TGTGGGGATCTCAGCCATAGT
	R	AGTGGGCTAAGACCCATCCTG
Resistin	F	CTGTCCAGTCTATCCTTGCACAC
	R	CAGAAGGCACAGCAGTCTTGA
PPARα	F	GCGTACGGCAATGGCTTTAT

	R	GAACGGCTTCCTCAGGTTCTT
PPARγ	F	GGAAAGACAACGGACAAATCAC
	R	TACGGATCGAAACTGGCAC
Otop1	F	ACTAGGACCCCGTCGAATCT
	R	ACCATGCTCTACGTGCTGTG
Cox7a1	F	CAGCGTCATGGTCAGTCTGT
	R	AGAAAACCGTGTGGCAGAGA
Cidea	F	TGCTCTTCTGTATCGCCCAGT
	R	GCCGTGTTAAGGAATCTGCTG
Apoe	F	CTGACAGGATGCCTAGCCG
	R	CGCAGGTAATCCCAGAAGC
Adipsin	F	CTACAAGCGATGGTATGATGTGC
	R	GGACCCAACGAGGCATTCT
Eif2s3y	F	ATCTTGTCCTCAACCTCAGACT
	R	TTCTTTAGCCTGGCTTTCTTTCA
Neuronatin	F	CACAGCCCATTGCGAGAAGT
	R	GGCAAGTGCTACTCTGACGA
Sncg	F	AAAGACCAAGCAGGGAGTAACG
	R	GACCACGATGTTTTCAGCCTC
Arxes2	F	AAAGTGGAAGACTTCACCGGA
	R	TCTGCCGACAGATAAAGGAAGA
Ces1d	F	GAGACCCAAGGCAGTAATAGGA
	R	GAGTTGAGGCACCAATCTTCA
Rasd1	F	GATGTGCCCAAGCGACTCT
	R	TGAGGAAGCGCGACACAAT
Psmb10	F	CACTGAGATGACTACGCGGAT
	R	GCTGAGGTCCGTTCAAATCAAC
Rgs2	F	GAGAAAATGAAGCGGACACTCT
	R	GCAGCCAGCCCATATTTACTG
Tfam	F	ATTCCGAAGTGTTTTTCCAGCA
	R	TCTGAAAGTTTTGCATCTGGGT
MyoD	F	CCACTCCGGGACATAGACTTG
	R	CCACTCCGGGACATAGACTTG
Myh1	F	GCGAATCGAGGCTCAGAACAA
	R	GTAGTTCCGCCTTCGGTCTTG
GABPα	F	GTAGTTCCGCCTTCGGTCTTG
	R	TCCTGCTCTTTTCTGTAGCCT
**lipolysis**		
Hsl	F	GGCTCACAGTTACCATCTCACC
	R	GAGTACCTTGCTGTCCTGTCC
Atgl	F	TCCCACTTTAGCTCCAAGGAT
	R	AGCTTCCTCTGCATCCTCTTC

Acsl1	F	TCTTGGTGTACTACTACGACGAT
	R	CGAGAACCTAAACAAGGACCATT
Acox1	F	CCGCCACCTTCAATCCAGAG
	R	CAAGTTCTCGATTTCTCGACGG
Human FOXP1	F	GGGGCAGTATGGACAGTGGATGA
	R	TTGAGAGGTGTGCAGTAGGCGTG
Human ADRB3	F	TTTGCCAACGGCTCGAC
	R	CGTCAGGTTCTGGAGGGTAG
Human B-ACTIN	F	CCAGCACAATGAAGATCAAGAT
	R	AGAAAGGGTGTAACGCAACTAA
**Genoytping**		
Foxp1-Floxed	F	CTCCTAGTCACCTTCCCCAGTGC3
	R	GAACACTGTCGAATGACCCTGC3
Cre	F	TTTCCCGCAGAACCTGAAGA
	R	GGTGCTAACCAGCGTTTTCGT
Ap2-Foxp1	F	ATAAGGAATAATGGGGGGAA
	R	TGTTGGAGAAGTTGAAGCTGCA
**Adrb3-shRNA**		
Adrb3-shRNA	F	taGGATCCggagTcacgTgcagtcaTcttGCAAGAGaagAtgactgcAcgtgActccTCTAGAta
	R	aTCTAGAggagTcacgTgcagtcaTcttCTCTTGCaagAtgactgcAcgtgActccGGATCCta
**ChIP-PCR**		
Adrb3 ChIP	F	aagcgaggattttcttcccact
	R	tccatctggcctccacatatag
**Adrb3-Luc**		
Adrb3-promoter	F	gagcgtctctgacagtcactgc
	R	ttacaattatggagtagcaacta

Fig. 1

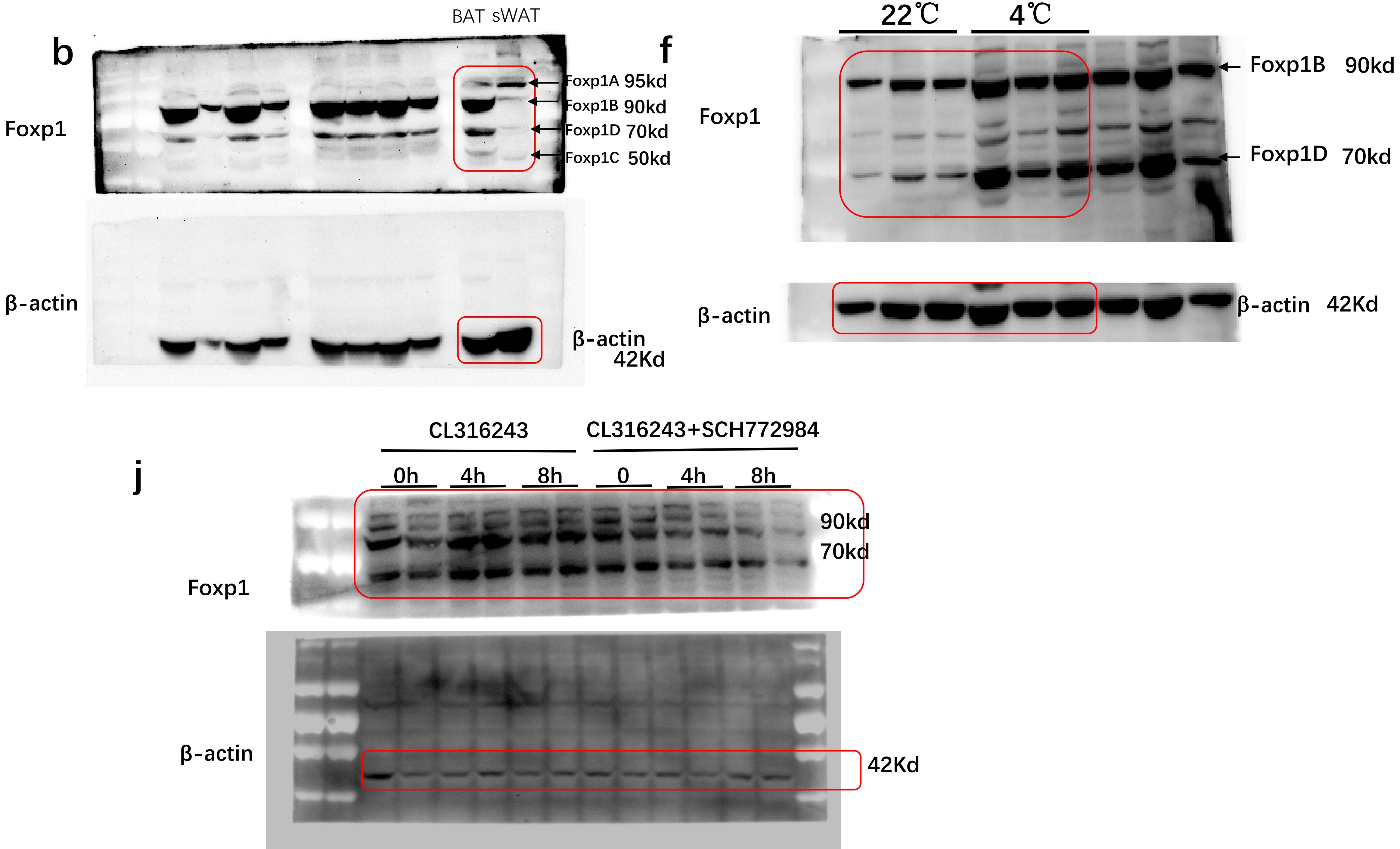
b
BAT sWAT
Foxp1
Foxp1A 95kd
Foxp1B 90kd
Foxp1D 70kd
Foxp1C 50kd
β-actin
β-actin
42Kd
f
22℃
4℃
Foxp1
Foxp1B 90kd
Foxp1D 70kd
β-actin
β-actin 42Kd
j
CL316243
CL316243+SCH772984
0h
4h
8h
0
4h
8h
90kd
70kd
Foxp1
β-actin
42Kd

Fig. 4

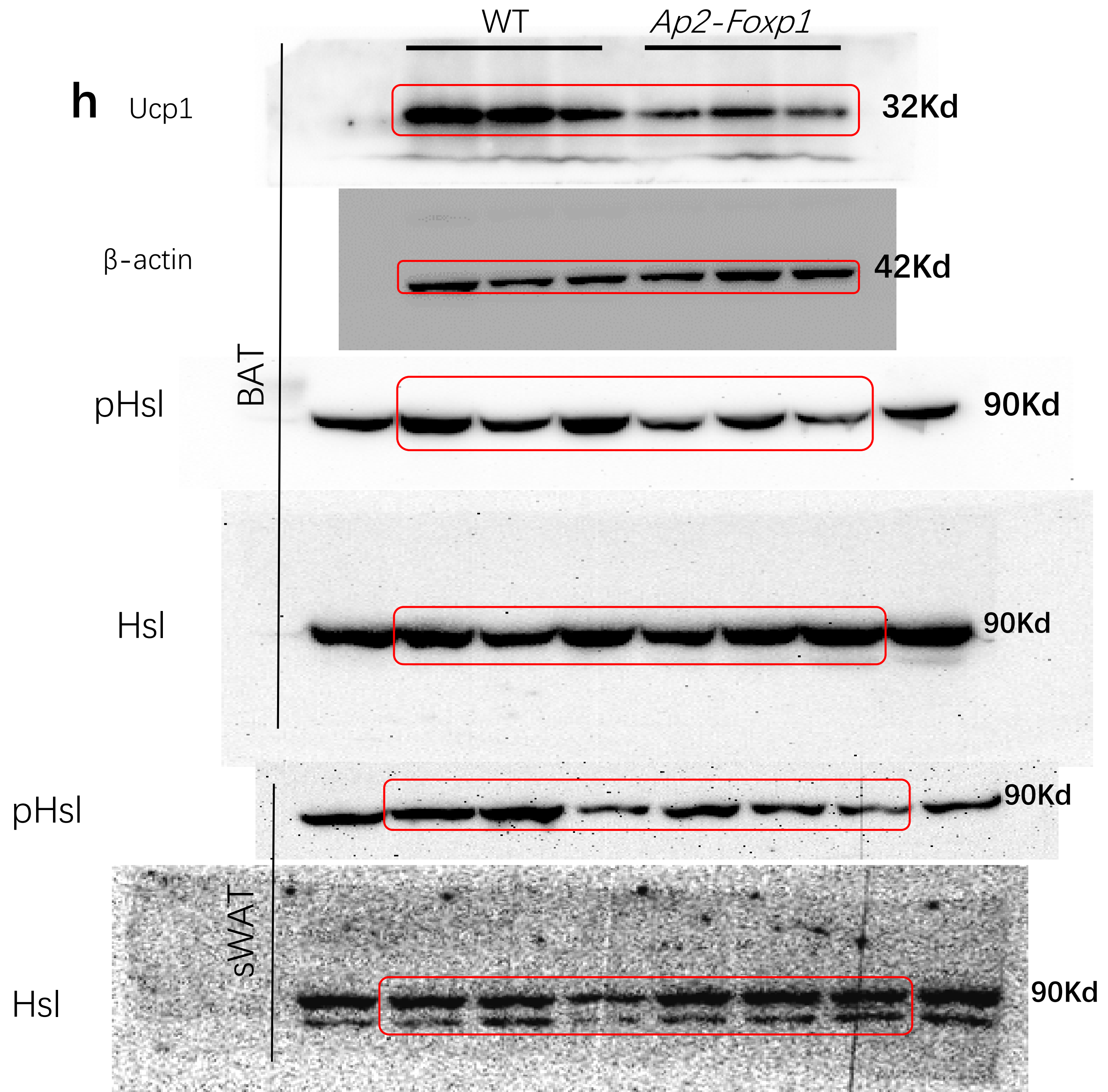
h
WT
Ap2-Foxp1
Ucp1
32Kd
β-actin
42Kd
pHsl
90Kd
Hsl
90Kd
BAT
pHsl
90Kd
Hsl
90Kd
sWAT

Fig. 5

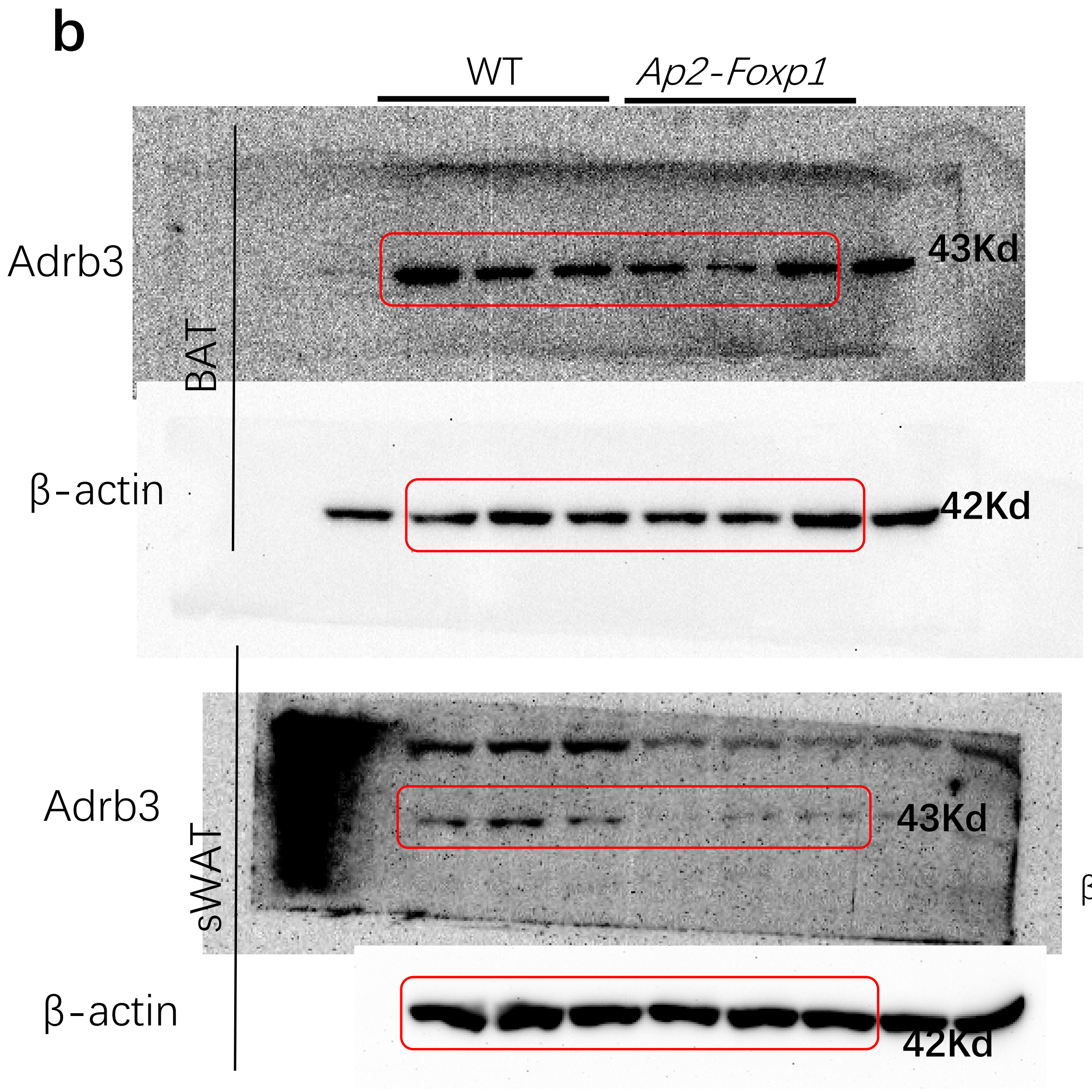

b
WT
Ap2-Foxp1
Adrb3
43Kd
BAT
β-actin
42Kd
Adrb3
43Kd
sWAT
β-actin
42Kd

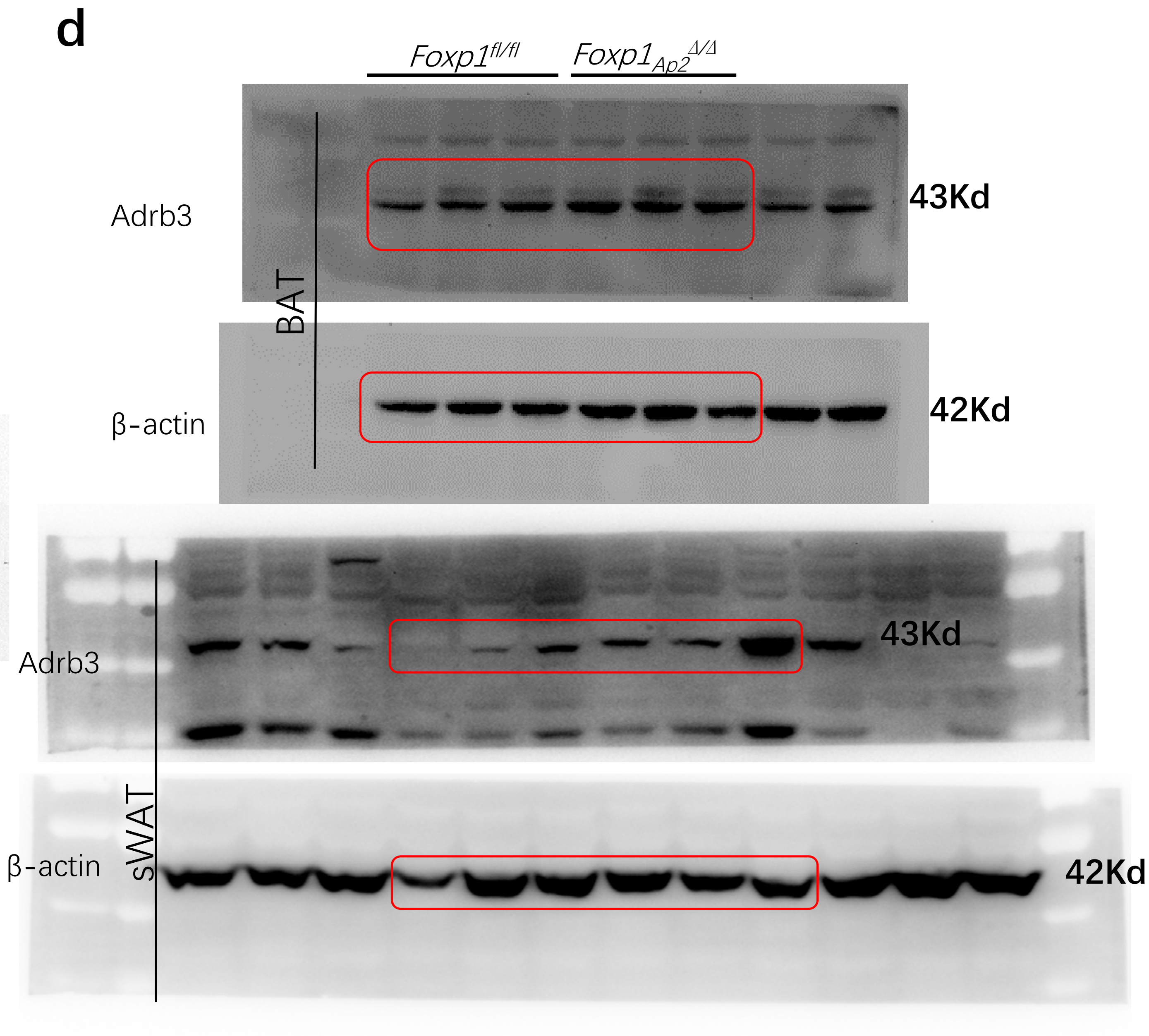

d
Foxp1fl/fl
Foxp1Ap2Δ/Δ
Adrb3
43Kd
BAT
β-actin
42Kd
Adrb3
43Kd
sWAT
β-actin
42Kd

Fig. 6

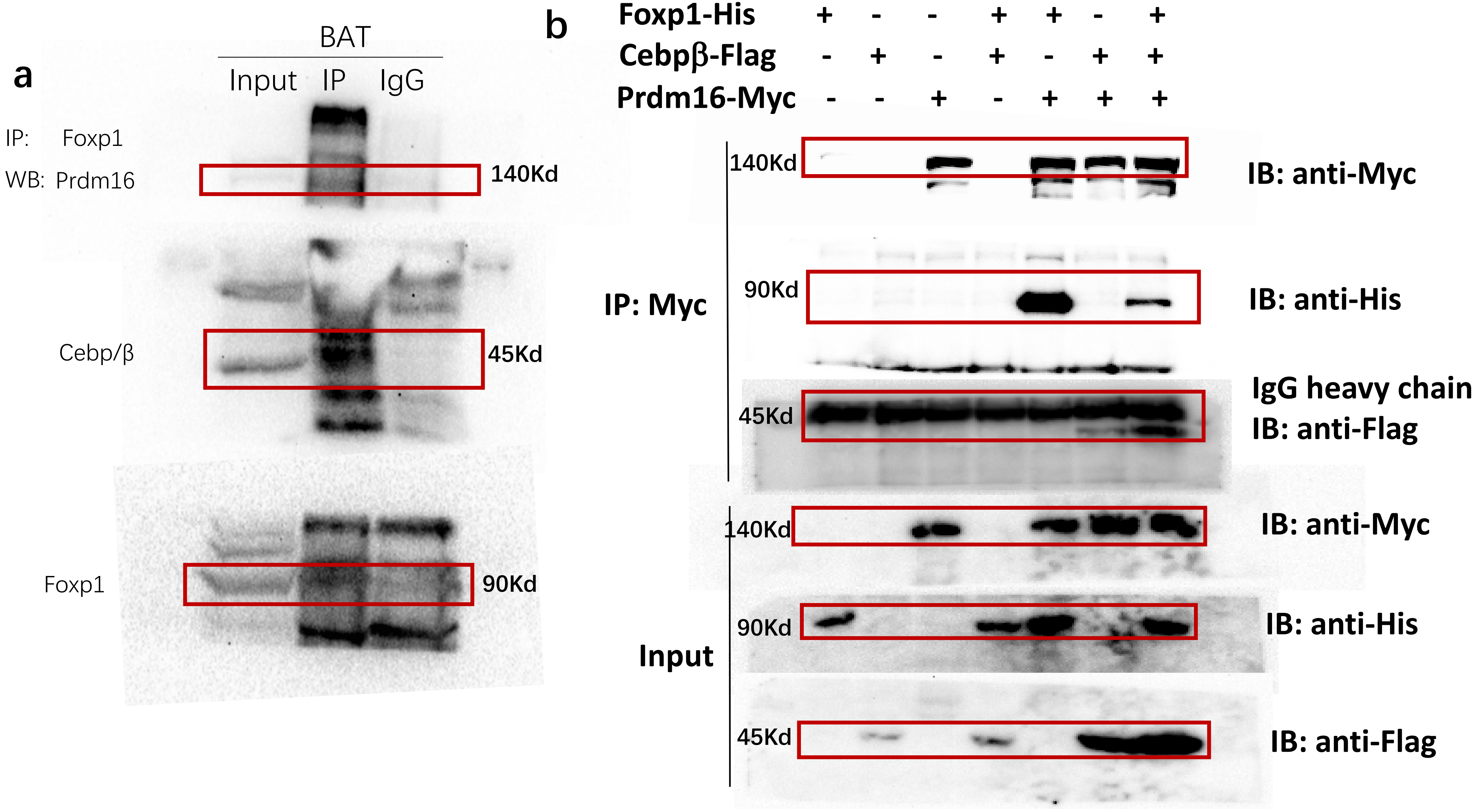
a
BAT
Input
IP
IgG
IP: Foxp1
WB: Prdm16
140Kd
Cebp/β
45Kd
Foxp1
90Kd
b
Foxp1-His + - - + + - +
Cebpβ-Flag - + - + - + +
Prdm16-Myc - - + - + + +
IP: Myc
140Kd
IB: anti-Myc
90Kd
IB: anti-His
45Kd
IgG heavy chain
IB: anti-Flag
Input
140Kd
IB: anti-Myc
90Kd
IB: anti-His
45Kd
IB: anti-Flag

Fig. S1

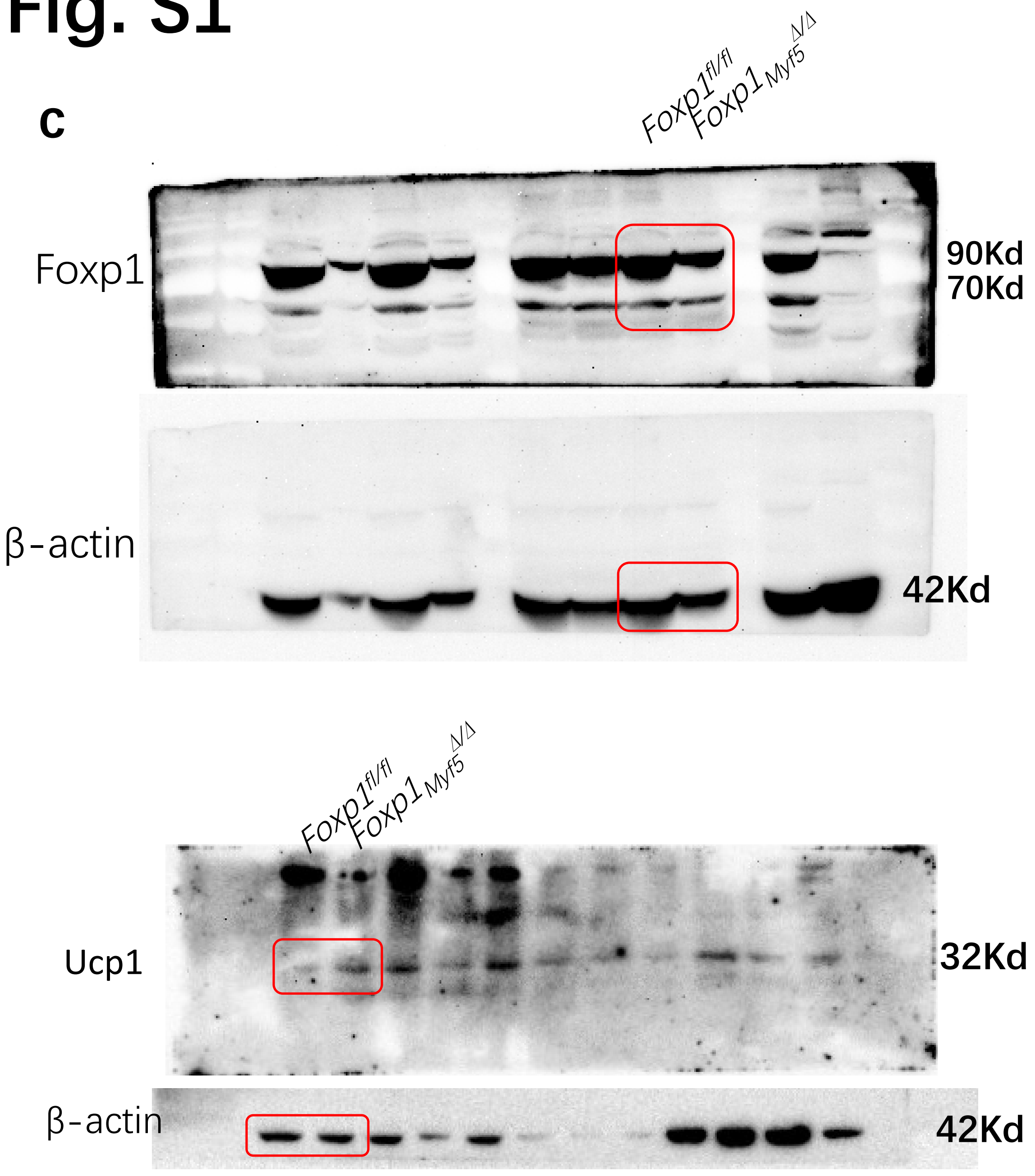
c
Foxp1$^{fl/fl}$
Foxp1$_{Myf5}^{\Delta/\Delta}$
Foxp1
90Kd
70Kd
β-actin
42Kd
Foxp1$^{fl/fl}$
Foxp1$_{Myf5}^{\Delta/\Delta}$
Ucp1
32Kd
β-actin
42Kd

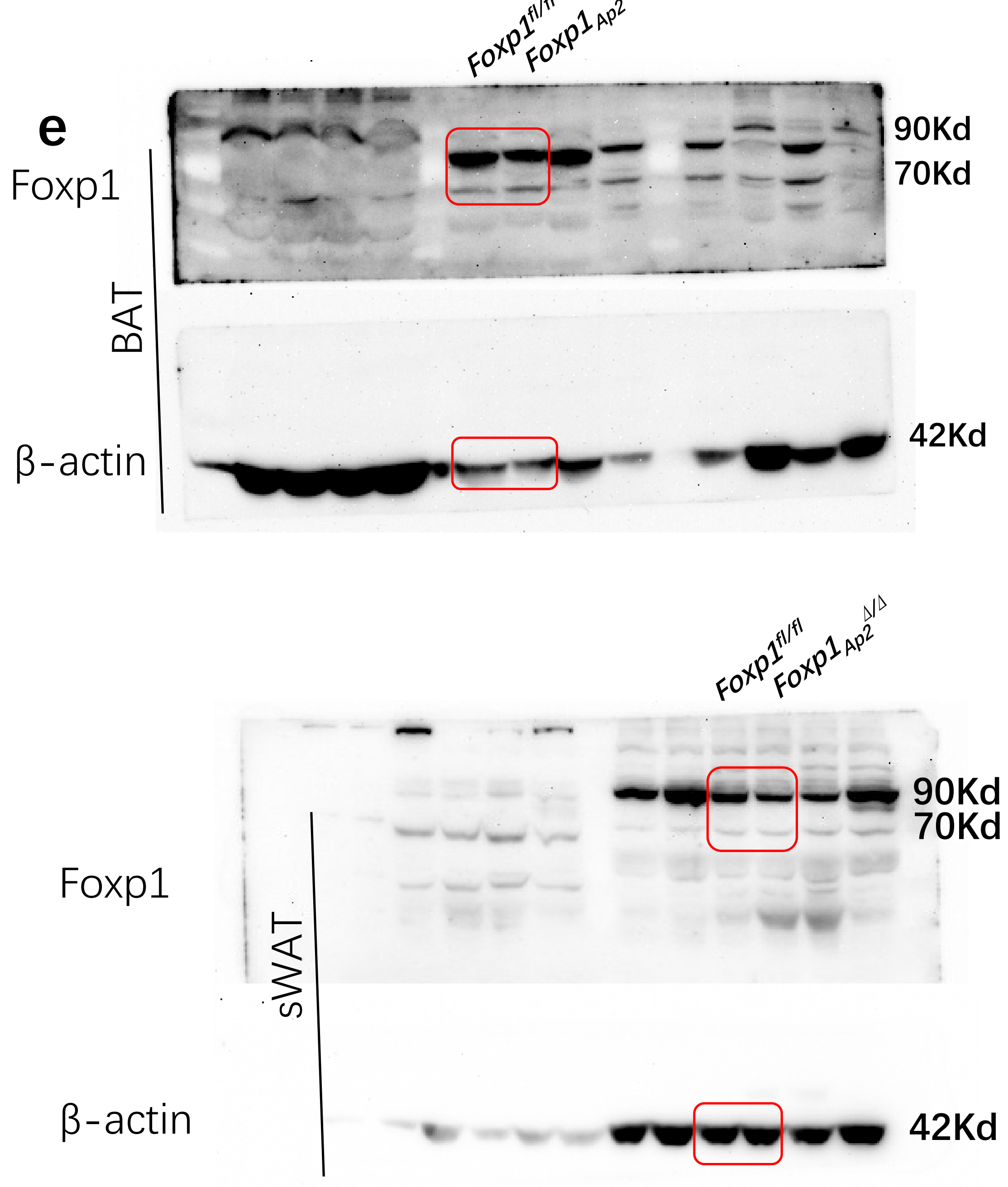
e
Foxp1$^{fl/fl}$
Foxp1$_{Ap2}$
Foxp1
90Kd
70Kd
BAT
β-actin
42Kd
Foxp1$^{fl/fl}$
Foxp1$_{Ap2}^{\Delta/\Delta}$
Foxp1
90Kd
70Kd
sWAT
β-actin
42Kd

Fig. S2

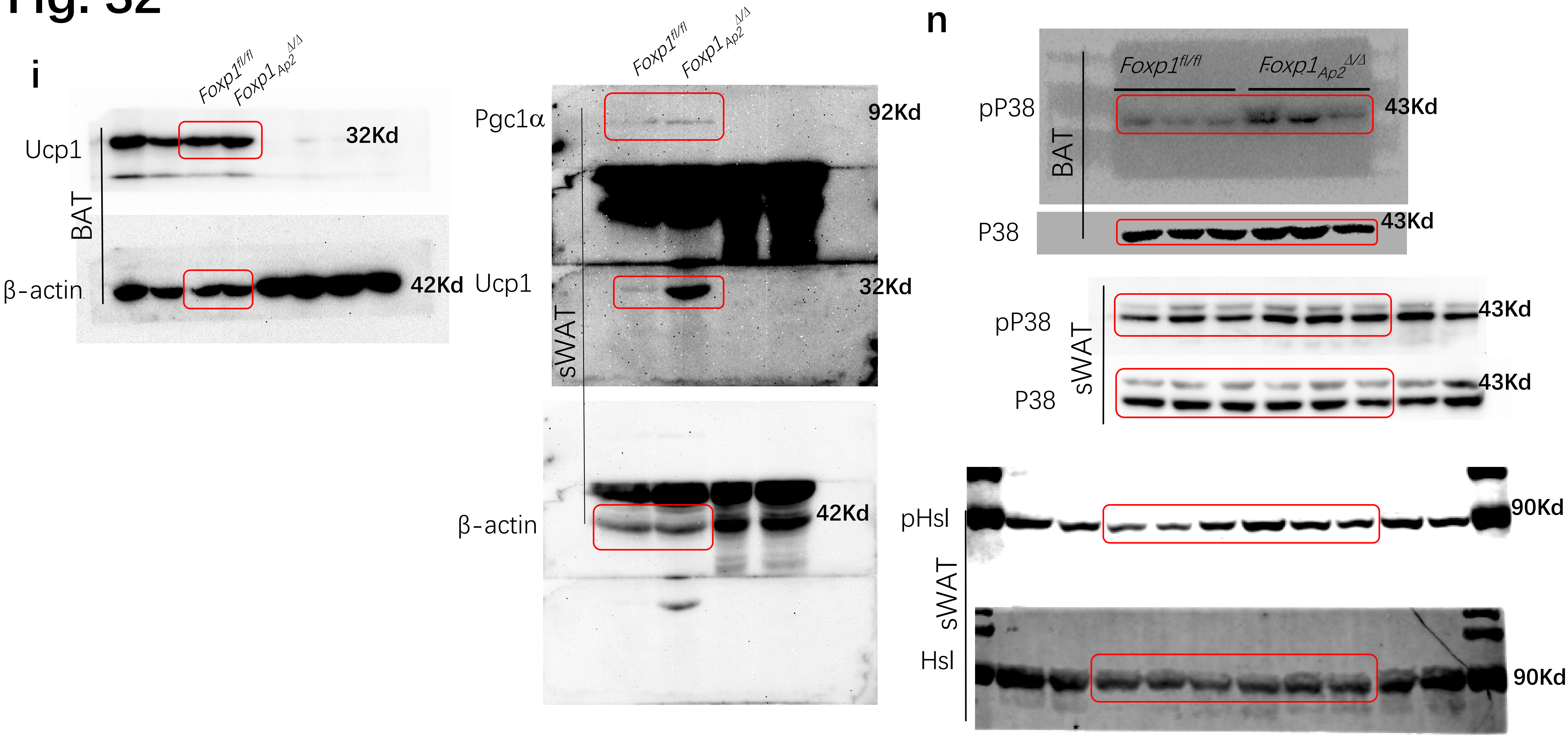
i
Foxp1fl/fl
Foxp1Ap2Δ/Δ
Ucp1
32Kd
BAT
β-actin
42Kd
Pgc1α
92Kd
Ucp1
32Kd
sWAT
β-actin
42Kd
n
Foxp1fl/fl
Foxp1Ap2Δ/Δ
pP38
43Kd
BAT
P38
43Kd
pP38
43Kd
sWAT
P38
43Kd
pHsl
90Kd
sWAT
Hsl
90Kd

Fig. S5

b

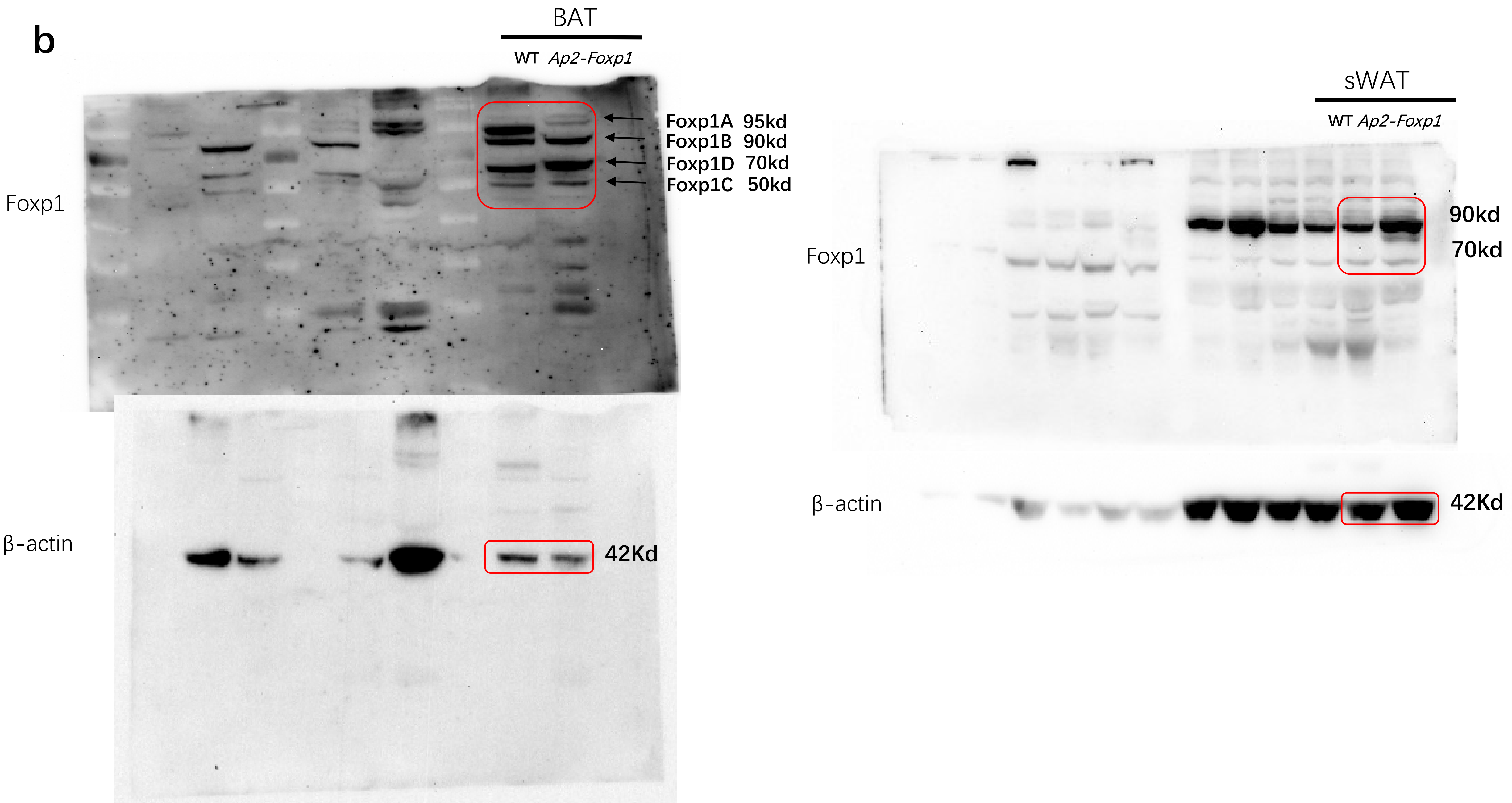
BAT
WT Ap2-Foxp1
Foxp1
Foxp1A 95kd
Foxp1B 90kd
Foxp1D 70kd
Foxp1C 50kd
β-actin
42Kd
sWAT
WT Ap2-Foxp1
Foxp1
90kd
70kd
β-actin
42Kd

Fig. S6

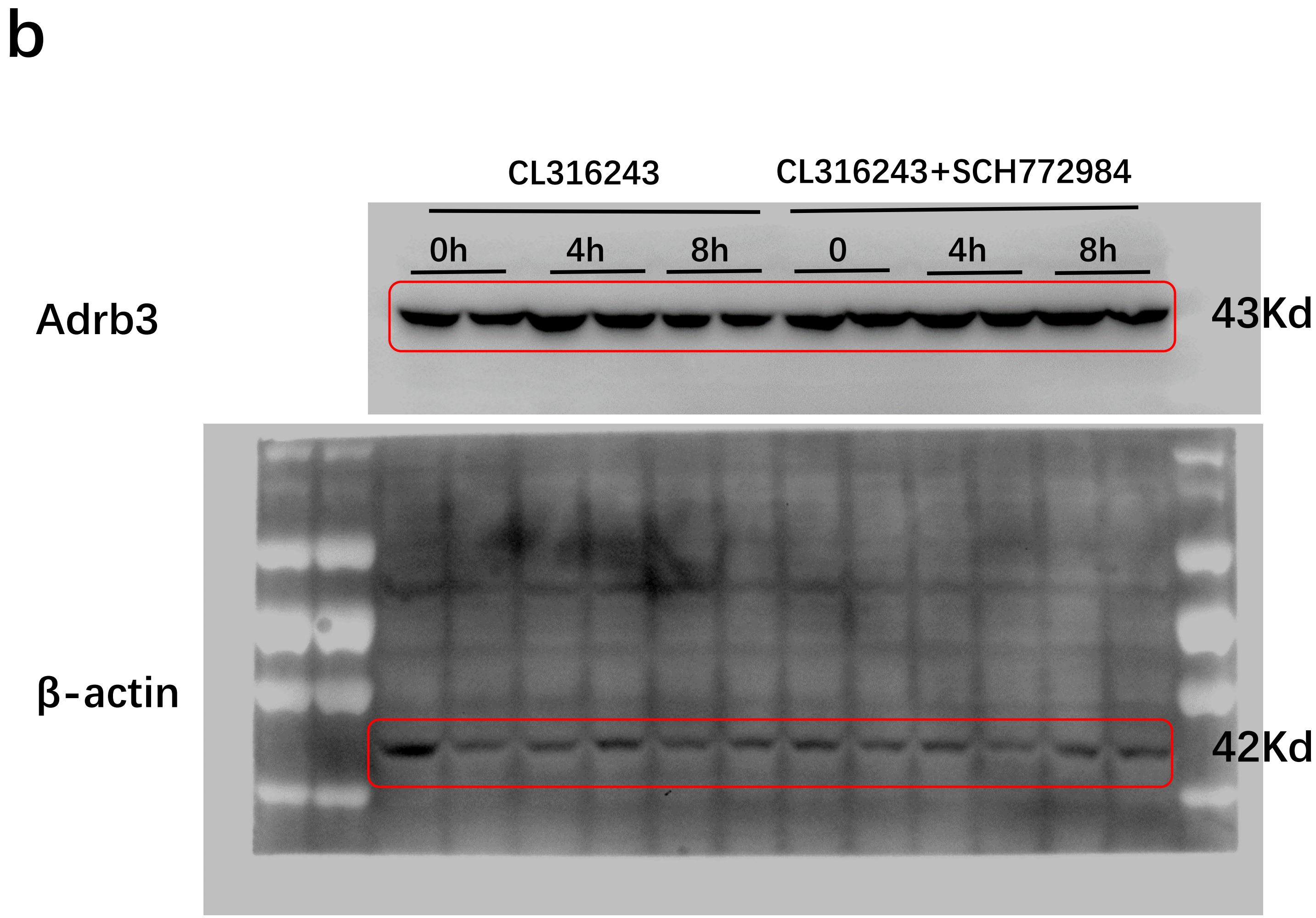
b
CL316243
CL316243+SCH772984
0h
4h
8h
0
4h
8h
Adrb3
43Kd
β-actin
42Kd
