# Supplementary material for: Foxp1 controls brown/beige adipocyte differentiation and thermogenesis through regulating β3-AR desensitization
Source: Nat Commun. 2019 Nov 7;10:5070. doi: 10.1038/s41467-019-12988-8 (PMC6838312; doi:10.1038/s41467-019-12988-8)
Supplement: Supplementary file 3 — Reporting Summary [file 41467_2019_12988_MOESM3_ESM.pdf]

nature research

Corresponding author(s): Xizhi Guo

Last updated by author(s): Jun 5, 2019

# Reporting Summary

Nature Research wishes to improve the reproducibility of the work that we publish. This form provides structure for consistency and transparency in reporting. For further information on Nature Research policies, see Authors & Referees and the Editorial Policy Checklist.

## Statistics

For all statistical analyses, confirm that the following items are present in the figure legend, table legend, main text, or Methods section.

| n/a | Confirmed | |
|---|---|---|
| ☐ | ☒ | The exact sample size (*n*) for each experimental group/condition, given as a discrete number and unit of measurement |
| ☐ | ☒ | A statement on whether measurements were taken from distinct samples or whether the same sample was measured repeatedly |
| ☐ | ☒ | The statistical test(s) used AND whether they are one- or two-sided<br>*Only common tests should be described solely by name; describe more complex techniques in the Methods section.* |
| ☐ | ☒ | A description of all covariates tested |
| ☒ | ☐ | A description of any assumptions or corrections, such as tests of normality and adjustment for multiple comparisons |
| ☐ | ☒ | A full description of the statistical parameters including central tendency (e.g. means) or other basic estimates (e.g. regression coefficient) AND variation (e.g. standard deviation) or associated estimates of uncertainty (e.g. confidence intervals) |
| ☐ | ☒ | For null hypothesis testing, the test statistic (e.g. *F*, *t*, *r*) with confidence intervals, effect sizes, degrees of freedom and *P* value noted<br>*Give P values as exact values whenever suitable.* |
| ☒ | ☐ | For Bayesian analysis, information on the choice of priors and Markov chain Monte Carlo settings |
| ☒ | ☐ | For hierarchical and complex designs, identification of the appropriate level for tests and full reporting of outcomes |
| ☒ | ☐ | Estimates of effect sizes (e.g. Cohen's *d*, Pearson's *r*), indicating how they were calculated |

*Our web collection on statistics for biologists contains articles on many of the points above.*

## Software and code

Policy information about availability of computer code

| | |
|---|---|
| Data collection | NO |
| Data analysis | NO |

For manuscripts utilizing custom algorithms or software that are central to the research but not yet described in published literature, software must be made available to editors/reviewers. We strongly encourage code deposition in a community repository (e.g. GitHub). See the Nature Research guidelines for submitting code & software for further information.

## Data

Policy information about availability of data

All manuscripts must include a data availability statement. This statement should provide the following information, where applicable:

- Accession codes, unique identifiers, or web links for publicly available datasets
- A list of figures that have associated raw data
- A description of any restrictions on data availability

The source data underlying Figs 1b, 1d-j, 2c-d, 2i-k, 3a-d, 3g-k, 4f-h, 4j, l, 5a-d, 5f, 5g, 5i, 5j, and 6a, 6b, 6d, 6e and Supplementary Figs 1a, S1c-e, 2e, 2g-j, 3b-d, 3f-j, 4a-d, 4f, 4i-l, 5 and 6 are provided as a Source Data file. All raw western blotting data could be found in the Supplementary Information. All relevant data are available from the authors.

# Field-specific reporting

Please select the one below that is the best fit for your research. If you are not sure, read the appropriate sections before making your selection.

☒ Life sciences ☐ Behavioural & social sciences ☐ Ecological, evolutionary & environmental sciences

For a reference copy of the document with all sections, see nature.com/documents/nr-reporting-summary-flat.pdf

# Life sciences study design

All studies must disclose on these points even when the disclosure is negative.

| | |
|---|---|
| Sample size | For qPCR and western blot, at least three samples were collected for tests. For cell differentiation, at least three different biological samples were collected. For metabolic analysis, usually 7-10 samples were required for each group. |
| Data exclusions | No data was excluded in statistics. |
| Replication | Each experiments were at least individually replicated three times. |
| Randomization | The experiments were not randomized. The data was collected according to the mice with each genotype. It does not affect the effectiveness of statistics. |
| Blinding | The investigators were not blinded to allocation during experiments or outcome assessments. The data was collected according to the mice with each genotype. It does not affect the effectiveness of statistics. |

# Reporting for specific materials, systems and methods

We require information from authors about some types of materials, experimental systems and methods used in many studies. Here, indicate whether each material, system or method listed is relevant to your study. If you are not sure if a list item applies to your research, read the appropriate section before selecting a response.

## Materials & experimental systems

| n/a | Involved in the study | |
|---|---|---|
| ☐ | ☒ | Antibodies |
| ☐ | ☒ | Eukaryotic cell lines |
| ☒ | ☐ | Palaeontology |
| ☐ | ☒ | Animals and other organisms |
| ☐ | ☒ | Human research participants |
| ☒ | ☐ | Clinical data |

## Methods

| n/a | Involved in the study | |
|---|---|---|
| ☐ | ☒ | ChIP-seq |
| ☒ | ☐ | Flow cytometry |
| ☒ | ☐ | MRI-based neuroimaging |

## Antibodies

| | |
|---|---|
| Antibodies used | Western blot primary antibodies: Foxp1 (Millipore, ABE68, 1:1000), C/ebpβ (Santa Cruz, sc-150, 1:500), Ucp1(Abcam, ab10893, 1 : 1000), PGC1a (Milipore, ab3242 1:1000), p38 (CST, 9212s, 1:1000), phosphorylated p38 (CST, 9211s, 1:1000), HSL (CST, 4107, 1:1000) and phosphorylated HSL (CST, 4126, 1:1000), β3-AR (mybioscience, MBS253490, 1:1000), His-Tag (MBL, M136-3, 1:2000), FLAG (Agilent, 200471, 1:2000) or β-actin (Selleck, A1016,1:2000)<br><br>Co-IP antibodies: Foxp1 (Millipore, ABE68, 1:100), Ucp1 (Abcam, ab10893, 1 : 50), Prdm16 (Abcam, ab106410, 1:50) |
| Validation | All the antibodies were validated for western blot or Co-IP in mouse samples. |

## Eukaryotic cell lines

Policy information about cell lines

| | |
|---|---|
| Cell line source(s) | HEK293T or 3T3-L1 cells |
| Authentication | The cells were authenticated by morphology and differentiated potency. |
| Mycoplasma contamination | All cell lines were tested to be negative for myoplasma contamination. |
| Commonly misidentified lines (See ICLAC register) | No |

## Animals and other organisms

Policy information about studies involving animals; ARRIVE guidelines recommended for reporting animal research

| | |
|---|---|
| Laboratory animals | The genetic backgrounds of all knockout mice were C57Bl/6J and the background of transgenic mice were 129S1/Sv. |
| Wild animals | NO |
| Field-collected samples | NO |
| Ethics oversight | All animal experiments were performed according to the guidelines (SYXK 2011-0112) and received ethical approval from committee of Bio-X Institutes of Shanghai Jiao Tong University. |

Note that full information on the approval of the study protocol must also be provided in the manuscript.

## Human research participants

Policy information about studies involving human research participants

| | |
|---|---|
| Population characteristics | Patients was clinically and pathologically diagnosed with pheochromocytoma, which is under adrenergic stress due to extremely excessive catecholamine expression . |
| Recruitment | Adult patients and sex-, age-, and BMI-matched control subjects were recruited by clinical doctors. |
| Ethics oversight | The human study was approved by the Institutional Review Board of Ruijin Hospital, Shanghai Jiao Tong University School of Medicine. Written informed consent was provided from each participant prior to inclusion in the study. |

Note that full information on the approval of the study protocol must also be provided in the manuscript.

## ChIP-seq

### Data deposition

☒ Confirm that both raw and final processed data have been deposited in a public database such as GEO.

☒ Confirm that you have deposited or provided access to graph files (e.g. BED files) for the called peaks.

| | |
|---|---|
| Data access links<br>*May remain private before publication.* | http://www.ncbi.nlm.nih.gov/bioproject/547458 |
| Files in database submission | ChIP-Foxp1_combined_R1.fastq<br>ChIP-Foxp1_combined_R2.fastq<br>ChIP-IgG_combined_R1.fastq<br>ChIP-IgG_combined_R2.fastq |
| Genome browser session<br>(e.g. UCSC) | No longer applicable |

### Methodology

| | |
|---|---|
| Replicates | 3 replicates of SVF cells in 10 cm plates were collected. |
| Sequencing depth | The sequence depth of Foxp1 ChIP data is 29.5M, total number of reads is 29492390, Unique mapped reads is 8986334. For control group, the sequence depth is 24M, total number of reads is 24018812, the number of unique mapped reads is 4803012. |
| Antibodies | anti-Foxp1 antibody, Millipore, Cat# ABE68 |
| Peak calling parameters | callpeak -f BAM -B --SPMR -g mm -p 0.05 --keep-dup auto --call-summits |
| Data quality | We obtained 14288 peaks with MACS software in total at P-value<0.05, of these, 56 peaks are significant at BHFDR<0.05 and above 5-fold enrichment(Benjamini Hochberg false discovery rate, BHFDR). |
| Software | Bowtie 2.2.6 ; MACS 2.1.1; Samtools 1.6 |
